# Functional response metrics explain and predict high but differing ecological impacts of juvenile and adult lionfish

**DOI:** 10.1098/rsos.240855

**Published:** 2024-08-21

**Authors:** Monica McCard, Nathan McCard, Neil E. Coughlan, Josie South, Louise Kregting, Jaimie T. A. Dick

**Affiliations:** ^1^ Institute for Global Food Security, School of Biological Sciences, Queen’s University Belfast, 19 Chlorine Gardens, Belfast BT9 5DL, UK; ^2^ Queen’s University Marine Laboratory, 12–13 The Strand, Portaferry BT22 1PF, UK; ^3^ School of Biological and Environmental Sciences, Liverpool John Moore University, Byrom Street, Liverpool L3 3AF, UK; ^4^ School of Biological, Earth and Environmental Sciences, University College Cork, Distillery Fields, North Mall, Cork, Ireland; ^5^ Water@Leeds, School of Biology, University of Leeds, Woodhouse, Leeds LS2 9JT, UK; ^6^ The New Zealand Institute for Plant and Food Research Ltd, Nelson, New Zealand

**Keywords:** invasive, functional response ratio, *Pterois volitans*, impact prediction, *Nephrops norvegicu*s

## Abstract

Recent accumulation of evidence across taxa indicates that the ecological impacts of invasive alien species are predictable from their functional response (FR; e.g. the maximum feeding rate) and functional response ratio (FRR; the FR attack rate divided by handling time). Here, we experimentally derive these metrics to predict the ecological impacts of both juvenile and adult lionfish (*Pterois volitans*), one of the world’s most damaging invaders, across representative and likely future prey types. Potentially prey-population destabilizing Type II FRs were exhibited by both life stages of lionfish towards four prey species: *Artemia salina*, *Gammarus oceanicus*, *Palaemonetes varians* and *Nephrops norvegicus*. FR magnitudes revealed ontogenetic shifts in lionfish impacts where juvenile lionfish displayed similar if not higher consumption rates than adult lionfish towards prey, apart from *N. norvegicus*, where adult consumption rate was considerably higher. Additionally, lionfish FRR values were very substantially higher than mean FRR values across known damaging invasive taxa. Thus, both life stages of lionfish are predicted to contribute to differing but high ecological impacts across prey communities, including commercially important species. With lionfish invasion ranges currently expanding across multiple regions globally, efforts to reduce lionfish numbers and population size structure, with provision of prey refugia through habitat complexity, might curtail their impacts. Nevertheless, the present study indicates that management programmes to support early detection and complete eradication of lionfish individuals when discovered in new regions are advised.

## 1. Introduction

Invasive species are predicted to continue to increase in number and severity of ecological impacts [1,2]. However, species-specific impacts are difficult to predict with surety and can vary considerably among invaded ecosystems due to a plethora of biotic and abiotic interactions (e.g. [3,4]). Also, invaders with highly similar morphological and behavioural traits, and even congeneric species, may differentially impact invaded regions (e.g. [5]). Thus, we require robust metrics that are both explanatory and predictive of ecological impact across habitats and trophic and taxonomic groups [6,7].

The red lionfish, *Pterois volitans*, is currently considered one of the most invasive and ecologically harmful marine fish worldwide [8], with populations spreading across the western Atlantic Ocean, Caribbean Sea [9] and Mediterranean Sea [10]. Both *P. volitans* and its sister species, *Pterois miles*, are also currently establishing themselves across the Mediterranean. Due to the significant impact of *P. miles* and the extensive invasion by *P. volitans*, we use *P. volitans* in this study as a proxy when referring to lionfish. As generalist and opportunistic predators, lionfish show dietary plasticity for prey items [11] and demonstrate clear negative impacts on some prey species [12]. Although adult lionfish are primarily piscivorous, they have also been recorded to predate on a variety of invertebrates (e.g. [13]). Most lionfish dietary studies have been completed on adult individuals due to ease of capture compared to juveniles (i.e. <15 cm; see [14–21]), and therefore juvenile lionfish predation is data limited; however, information available suggests a predominance of small crustaceans and larval fish [17,22,23]. As a consequence of culling programmes, selective removal of large individuals tends to occur in invaded areas, with the remaining population being largely composed of juveniles and smaller-sized individuals which are cryptic, fast moving and avoid culling activity by spear fishers [24,25]. Accordingly, a predictive assessment of lionfish invasion impacts thus requires dual examination of juvenile and adult feeding rates on representative and future likely prey communities.

Functional response (FR) metrics (described as Type I, Type II and Type III) have been used successfully to assess the ecological impact of current, future and emerging invasive species, through consideration of resource consumption (i.e. the amount of a resource utilized, such as prey) as a function of resource density [3,4,6]. The Type I response is a linear response, characteristic of filter feeders which are not constrained by handling times [26]; density-dependent Type II responses are characterized by a plateauing of consumption as prey density increases [27]; Type III responses are represented by a sigmoidal curve due to a reduction of consumption at low prey densities, often providing low-density refugia to rare prey species and sometimes related to learned predator avoidance behaviour in prey populations [28,29]. Deriving the type of FR, plus the attack rates, handling times and maximum feeding rates, has been successful across taxa in explaining and predicting invader impacts [4,6,30]. However, while high attack rates and low handling times predict high impact [7], predictions based on either parameter alone can be contradictory (e.g. when handling times are low, but attack rates are also low [31]). To resolve this, Cuthbert *et al*. [31] proposed a composite metric, the functional response ratio (FRR), that is, attack rate divided by handling time. The FRR has a clear pattern of high values predicting high ecological impact, where the worst invaders have an FRR mean of 83.36. This benchmarking allows comparison of newly derived FRRs, and hence likely ecological impact, in studies such as the present regarding lionfish (see [31]). FRR, however, is applicable for Type II and Type III comparison as Type I FR is devoid of a handling time.

We thus assessed the predatory impacts of both juvenile and adult *P. volitans* on four prey species by employing the FR metrics approach. The selected prey species were used to mimic a host of similar prey found across the invaded and future ranges of lionfish, namely the brine shrimp (*Artemia salina*), marine gammarid (*Gammarus oceanicus*)*,* dwarf white shrimp (*Palaemonetes varians*) and finally Dublin Bay prawn (*Nephrops norvegicus*), as the lionfish range is currently expanding across the Mediterranean (albeit predominantly *P. miles*) into areas where commercially and ecologically valuable *N. norvegicus* are located [32]. Given that morphological and metabolic changes may affect diet, leading to variations in preferred prey types, as well as possible restricted ability of juvenile lionfish to predate larger prey due to the size relationship between predator and prey. We hypothesized that juvenile and adult lionfish will display FR metrics consistent with high ecological impact, with ontogenetic shifts in these metrics due to predator/prey size.

## 2. Material and methods

### 2.1. Animal collection and maintenance

Experiments were undertaken at Queen’s University Marine Laboratory (QML), Portaferry, UK, between January and October 2019. Juvenile *P. volitan* lionfish (*n* = 8) and adult *P. volitan* lionfish (*n* = 8) were obtained from Seahorse Aquarium, Dublin. Juveniles had a total body length (mean ± s.e.) of 102.80 ± 3.18 mm, with a pectoral fin diameter of 57.89 ± 4.80 mm, as measured across the widest point when elongated. Adults measured 305.51 ± 3.73 mm in length with a pectoral fin diameter of 265.51 ± 6.37 mm. Juveniles were kept in a holding tank (W: 32 cm × L: 152 cm × H: 45 cm, 218 l) with external filtration containing UV- and sand-filtered recirculated Strangford Lough seawater. Adult holding tanks separately employed the same filtration set-up, while two adults were housed per tank (W: 82 cm × L: 227 cm × H: 61 cm, 1130 l). Up to 10% of the tank water was changed daily, monitoring temperature, and conditions including pH and ammonium. Seawater was maintained at 25.0 ± 1.0°C using an aquarium heater under a natural light regime. Lionfish were maintained daily ad libitum on frozen anchovy to avoid predator learning behaviour of the experimental prey species. Feeding experiments were conducted within glass tanks (juveniles: W: 33 cm × L: 46 cm × H: 30 cm, 45 l; adults: W: 51 cm × L: 132 cm × H: 38 cm, 250 l) maintained at 25.0 ± 1.0°C to ensure lionfish welfare. Experimental tanks were scaled to reflect the difference between juvenile and adult lionfish when pectoral fins were fully elongated during feeding trails, where adults were approximately five times the size of juvenile lionfish. All fish were acclimated in the experimental arenas for a 30 min period immediately prior to experimentation.

Brine shrimp (*A. salina*), marine gammarid (*G. oceanicus*), dwarf white shrimp (*P. varians*) and Dublin Bay prawn (*N. norvegicus*) were used as live prey. *Artemia salina* were obtained from Seahorse Aquariums, Dublin, Ireland, while *G. oceanicus* and *P. varians* were obtained from Grosvenor Tropicals, Lisburn, UK. *Nephrops norvegicus* were caught in fishing grounds off the western Irish Sea, by the FV Fulmar, an 11.33 m trawler using a SELTRA in single-rig configuration. Once samples were landed, they were immediately brought to QML. *Artemia salina*, *G. oceanicus* and *P. varians* were maintained under identical conditions to hose for the predators in separate holding tanks (W: 15 cm × L: 20 cm × H: 18 cm, 10 l), whereas *N. norvegicus* were housed in a dark outdoor holding tank (H: 94 cm × W: 142 cm × L: 211 cm, 2800 l), which included tunnels for refuge. All prey species were acclimated to lionfish maintenance temperature of 25.0 ± 1.0°C before being introduced to the testing tank. *Artemia salina*, *G. oceanicus*, and *P. varians* were kept at 22.0 ± 1.0°C, which was then raised to 25.0 ± 1.0°C 60 min before introduction. In contrast, *N. norvegicus* was initially kept at 18.0 ± 1.0°C and gradually exposed to a temperature change in the testing tank over 60 min until reaching 25.0 ± 1.0°C. Intraspecific prey size was standardized throughout all trials. Total length (mean ± s.e.): *A. salina*, (6.2 ± 0.8 mm); *G. oceanicus*, (10.7 ± 0.9 mm); *P. varians*, (11.3 ± 0.5 mm); and total carapace length for *N. norvegicus*, (20.1 ± 3.1 mm).

Selected prey species mimic those that are commonly found in lionfish stomachs across their invaded and potential future regions and have been used in previous lionfish FR experiments using similar laboratory set-ups to the present study [13,17,30,33–36]. The present study represented the first comparative assessment of juvenile and adult lionfish, as well as the first assessment of lionfish impact on *N. norvegicus*, which are a valuable commercial fishery species across the United Kingdom and European Union that will likely be threatened by the expanding lionfish invasion [37–39]. Furthermore, *N. norvegicus* can be used as a proxy for juveniles of other large crustacean species such as the Caribbean spiny lobster (*Panulirus argus*), the European lobster (*Homarus gammarus*) and anomurans (squat lobsters).

### 2.2. Functional response procedure

Each prey species was separately supplied at 15 densities (2, 4, 6, 8, 12, 16, 20, 25, 30, 35, 40, 45, 50, 55, 60; experiment replication *n* = 8 per density for each of the four prey species) in a randomized pattern of both prey species and densities. This was achieved with the re-use of the available lionfish in the following manner: following the addition of the allotted prey to the experimental tanks that contained an individual predator, FR experiments were initiated. Lionfish were allowed to feed for 3 h before being removed for enumeration of prey consumed. In a one month period, there were eight experiment days, with all lionfish being used on each experiment day (adult *n* = 8; juvenile *n* = 8). This facilitated a 3 day recovery period between experiment days. Re-use of individuals was essential due to the limited number of lionfish available, hence the recovery period (see [40]). The entire experiment was conducted over a 10 month period, with all lionfish being systematically exposed to all prey items at all densities, in a randomly allocated order. Controls consisted of one replicate of each prey type across all densities in the absence of lionfish predators.

### 2.3. Statistical analyses

Statistical analyses were undertaken using the ‘frair’ package in R [41]. Logistic regression was used to derive FR types based on analyses of proportional prey consumption across prey densities, with ‘prey density’ included as a continuous variable [42]. To model the FRs, data were fit using Rogers’ random predator equation, as prey were not replaced once consumed [43]:


(2.1) $$N_{e}=\;N_{0}\left(1-\exp\left(a\left(N_{e}h-T\right)\right)\right).$$

Wherein *N*_e_ represents the amount of prey consumed, *N*_0_ is initial prey density, *a* is the attack rate parameter, *h* is the handling time and *T* is the total time available. Data of prey eaten were then non-parametrically bootstrapped (*n* = 2000) to produce 95% confidence intervals (CIs) using initial maximum-likelihood estimates of *a* and *h*. The handling time parameter was used to determine maximum feeding rates (1/*h*) of lionfish across prey groups. Additionally, the FRR was calculated for each prey species using the parameter estimates of *a* and *h* derived from the FR curve from equation (2.1):


(2.2) FRR=a/h.

## 3. Results

Across all control groups (i.e. no predator) for all prey species, survival of the prey exceeded 99% in the absence of lionfish; therefore, all mortality of prey in experimental groups was assumed to be due to predation by lionfish.

### 3.1. Functional responses

**Figure 1.**
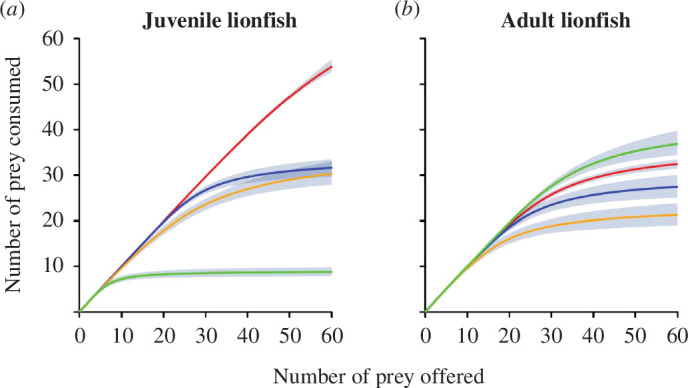
FRs showing the order of highest to lowest consumption of prey with both juvenile (*a*) and adult (*b*) *P. volitans* towards all prey types: *A. salina* (red), *G. oceanicus* (orange), *P. varians* (blue) and *N. norvegicus* (green). Shaded areas are bootstrapped (*n* = 2000) 95% CIs.

First-order terms were significantly negative as per Juliano [44], indicating Type II FRs by all lionfish towards all prey species (table 1; figure 1).

**Table 1.** First-order terms from logistic regression of prey consumed, alongside rounded initial and bootstrapped (*n* = 2000; 95% CIs) FR parameters (attack rates, handling times, maximum feeding rates and FRRs), of juvenile and adult *P. volitans* with different prey types.

predator	prey	first-order term, *p*	FR type	*a*, 95% CI	*h*, 95% CI	maximum feeding rate (1/*h*)	FRR (*a*/*h*)
juvenile *P. volitans*	*A. salina*	−0.09, <0.001	II	7.11, 5.47–9.43	0.01, 0.01–0.02	79.37	564.29
juvenile *P. volitans*	*G. oceanicus*	−0.06, <0.001	II	4.44, 3.18–6.14	0.03, 0.02–0.03	35.71	158.54
juvenile *P. volitans*	*P. varians*	−0.08, <0.001	II	10.15, 7.22–14.55	0.03, 0.03–0.03	34.48	350.14
juvenile *P. volitans*	*N. norvegicus*	−0.06, <0.001	II	6.45, 3.85–11.84	0.11, 0.10–0.13	8.93	57.62
adult *P. volitans*	*A. salina*	−0.07, <0.001	II	6.42, 5.19–7.94	0.03, 0.03–0.03	37.04	237.74
adult *P. volitans*	*G. oceanicus*	−0.06, <0.001	II	5.27, 3.61–7.83	0.04, 0.04–0.05	23.26	122.61
adult *P. volitans*	*P. varians*	−0.07, <0.001	II	6.86, 5.11–10.07	0.03, 0.03–0.04	30.30	207.76
adult *P. volitans*	*N. norvegicus*	−0.08, <0.001	II	7.15, 5.60–9.27	0.02, 0.02–0.03	43.48	310.91

#### 3.1.1. Juvenile lionfish

The attack rates and handling times of juvenile lionfish towards the four prey species resulted in FR magnitudes greatest for *A. salina*, lowest for *N. norvegicus* and intermediate for *G. oceanicus* and *P. varians* (table 1; figure 1). FRR values for juvenile lionfish were greatest for *A. salina* (564.29), followed by *P. varians* (350.14), *G. oceanicus* (158.54), and *N. norvegicus* (57.62: table 1). The first three FRR values are markedly higher than the mean FRR value of 83.36 that was found for highly damaging invaders across taxa (see [31]), by factors of 7, 4.5 and 2, predicting high ecological impacts of juvenile lionfish on prey with similar traits to *A. salina*, *P. varians* and *G. oceanicus*.

#### 3.1.2. Adult lionfish

The attack rates and handling times of adult lionfish result in quite different FR magnitudes compared with juveniles, with adult FRs ordered greatest for *N. norvegicus* followed by *A. salina*, *P. varians* and *G. oceanicus* (table 1; figure 1). FRR values for adult lionfish were greatest for *N. norvegicus* (310.91), then *A. salina* (237.74), *P. varians* (207.76) and *G. oceanicus* (122.61; table 1). All these FRR values are substantially higher than the mean FRR value of 83.36 found by Cuthbert *et al*. [31] by factors of 3.8, 3, 2.6 and 1.5, predicting high ecological impacts of adult lionfish on such crustacean prey.

## 4. Discussion

The observed consumption patterns and FR metrics predict that both juvenile and adult lionfish can exert damaging impact on a range of crustacean prey where larger individuals show size-dependent preferences for larger prey items compared to the gape-limited juveniles [13,45,46]. These findings are in line with *in situ* stomach content analyses (e.g. [15,17,23]).

The Type II FRs by both juveniles and adults suggest potential destabilizing effects on invertebrate prey species populations, although lionfish may switch prey preference for species that are more abundant in the environment, which provides a low-density prey refuge, and complex habitat structure can mitigate impacts [29,36]. These mitigating drivers may explain the difference in field impact between the Bahamas (high) and Belize (low) [47,48]. Habitat structure can offer refuges for prey, reducing predator search success at low prey densities, leading to sigmoid Type III FRs [49,50]. In contrast, the absence of habitat structure and the effects of arena size often result in Type II FRs [51]. Our comparative laboratory study used standardized conditions for all organisms without additional habitat complexity. Despite this, FR analyses and related impact assessment metrics are highly predictive of *per capita* impacts and simple laboratory settings of FR analyses are predictive of actual field impacts [6,7,52]. While lionfish use the entire water column to hunt, benthic and pelagic prey can attempt escape in both the upward and downward direction, which adds additional complexity to *in situ* foraging by lionfish in nominal two-dimensional benthic and three-dimensional pelagic environments.

Further predictive confidence of high lionfish impact is their remarkable FRR values, that were up to seven times higher than the mean FRR across known damaging invasive taxa [31]. The benchmark FRR values of Cuthbert *et al*. [31] indicate that, overall, mean FRRs of 83.36 typify high-impact invaders, since attack rates are high and handling times are low. The FRR values found here for lionfish were distinctively high, indicating an ecologically damaging ability of lionfish to find, subdue, consume and digest prey, which is clearly commensurate with actual field impacts of lionfish.

Lionfish pose a threat to commercial crustacean fisheries and can disturb benthic food webs in both current and potential invasion areas [53,54]. Lionfish have been observed at varying depths in regions they have infiltrated: surpassing 100 m in the Bahamas [55], reaching depths of 250 m in Honduras [56] and descending as far as 304 m in Bermuda [56] where dense lionfish populations have been identified at specific locations, particularly at or below 60 m, in select Bermuda sites [57,58]. The potential for high consumption rates of *N. norvegicus* by adult lionfish is a cause for concern considering that lionfish range expansions will overlap with commercially important fishing grounds. While juveniles also fed on *N. norvegicus*, they did so in smaller numbers, which may be due to limitations in gape and their inability to efficiently predate the hard exoskeleton [46,59]. *Nephrops norvegicus* are generally a deep-water species which reside in mud-flat burrows at depths of 20–800 m; given that lionfish have been found at mesophotic depths this suggests that deep-water populations could be sustained on *N. norvegicus* [60]. Furthermore, high FRR values on *P. varians* indicates high potential for consumption of functional analogue species such as *Pandalus montagui* and *Cragon crangon*, both of which form a high percentage of diet for native fish predators [61]. The full and pernicious impacts of lionfish invasion may be further revealed if prey depletion leads to trophic cascades through loss of prey for native predators [62,63].

While *A. salina* and *P. varians* are generally pelagic with high mobility [64,65], *G. oceanicus* and *N. norvegicus* tend to be epibenthic with a relatively lower rate of mobility [66–68]. It appears that both lionfish life stages can exploit pelagic and epibenthic prey [69,70], with utilization of prey items being linked to body type [15,71], size [69,72] and digestibility [73,74] rather than mobility [74] for juvenile lionfish, while adults appeared to better utilize the largest prey and least mobile prey. Previous studies have shown lionfish may specialize on small prey species that are solitary, nocturnal and bottom dwelling [34,69]; however, the adult lionfish in this study showed a reduced consumption of small epibenthic species (*G. oceanicus*) compared with the larger *N. norvegicus* and the pelagic species. This may indicate some difficulty in consumption within the tank confines due to spatial limitations on manoeuvrability.

The current geographical spread and increased growth in abundance of lionfish in the Atlantic have made eradication impossible [75]. While the data in this study were collected in a laboratory setting, with prey being presented in isolation from other prey resources, this work provides a basis for estimates of consumption rates of both juvenile and adult lionfish on representative and future prey types. Further, current management strategies for lionfish populations rely on the removal of adults [75,76]; however, our data indicate that juvenile lionfish can have a greater impact on native prey species than adult lionfish. This increased predation pressure reduces prey availability for native predators. Therefore, populations composed of juveniles and adults will have wide ranging impacts across multiple prey species, potentially driven by ontogenetic shifts in functional morphology, since juveniles have traits associated with a mechanical advantage during prey capture, whereas adult morphology is more associated with locomotion and sustained swimming but a lower suction velocity [46]. Accordingly, management strategies will need to be developed to efficiently control all life stages, rather than adults alone. Finally, while culling might reduce lionfish impacts through reduced numbers of individual predators, imaginative strategies to alter FR metrics might also be employed; for example Type III FRs and lowered FRRs may result from increased habitat complexity such as artificial reefs. The present study demonstrates both juvenile and adult lionfish can have a considerable impact on prey populations if allowed to establish and persist in regions at risk of invasion. Ultimately, early detection and eradication remain the best, if least utilized, strategies for invasive species management.

## Data Availability

The data are available from the Dryad Digital Repository [77].
